# STOP SHOUTING AT ME: The Influence of Case and Self-Referencing on Explicit and Implicit Memory

**DOI:** 10.3389/fpsyg.2021.685756

**Published:** 2021-06-09

**Authors:** George O. Ilenikhena, Haajra Narmawala, Allison M. Sklenar, Matthew P. McCurdy, Angela H. Gutchess, Eric D. Leshikar

**Affiliations:** ^1^Department of Psychology, University of Illinois at Chicago, Chicago, IL, United States; ^2^Department of Psychology, Brandeis University, Waltham, MA, United States

**Keywords:** self-reference, explicit memory, implicit memory, item memory, context memory

## Abstract

Evidence suggests that physical changes in word appearance, such as those written in all capital letters, and the use of effective encoding strategies, such as self-referential processing, improves memory. In this study we examined the extent both physical changes in word appearance (case) and encoding strategies engaged at study influence memory as measured by both explicit and implicit memory measures. Participants studied words written in upper and lower case under three encoding conditions (self-reference, semantic control, case judgment), which was followed by an implicit (word stem completion) and then an explicit (item and context) memory test. There were two primary results. First, analyses indicated a case enhancement effect for item memory where words written in upper case were better remembered than lower case, but only when participants were prompted to attend to the case of the word. Importantly, this case enhancement effect came at a cost to context memory for words written in upper case. Second, self-referencing increased explicit memory performance relative to control, but there was no effect on implicit memory. Overall, results suggest an item-context memory trade-off for words written in upper case, highlighting a potential downside to writing in all capital letters, and further, that both physical changes to the appearance of words and differing encoding strategies have a strong influence on explicit, but not implicit memory.

## Introduction

The current era is dominated by technology, where communication heavily involves written texts (texting, email, etc.) to relay information to one another. Because digital text messages have limited capacity to convey tone, people sometime use strategies to show emphasis in written communication, such as writing messages in all capital letters (i.e., all caps). Experimental evidence has shown that variations in the physical appearance of text, which is also known as *typographical cueing*, can affect the memorability of that information. Specifically, several studies have demonstrated that using typographic cues (e.g., all caps, bolding, italicizing) results in enhanced memory for the cued information (Crouse and Idstein, 1972; Foster and Coles, 1977; Golding and Fowler, 1992; Lorch et al., 1995; Dyson and Gregory, 2002). For instance, Lorch et al. (1995) evaluated the effects of capitalization on memory. In that study, participants read a 4-page, 2400-word text discussing topics related to energy and conservation. “Target” sentences were shown either as the only text written in all caps, or in control condition where the entire passage, including target and non-target sentences, were capitalized. Results demonstrated a *case enhancement effect* where recall was better for the capitalized texts compared to control. Although these studies have demonstrated that capitalization improves memory for capitalized information, this type of work used experimental designs that involve embedding short capitalized texts in longer passages of non-capitalized text (Schulman, 1974; Foster and Coles, 1977; Lorch et al., 1995). This experimental approach is problematic because the observed improvement in memory could simply be due to the distinctiveness of the capitalized passages (Hunt and Elliot, 1980; Fabiani and Donchin, 1995; Hunt, 1995), and not due to capitalization, *per se*. Thus, a better test of the influence of capitalization on memory would be a carefully controlled experiment where half of the materials are presented in upper case and half in lower case. We do so in this experiment.

In addition to physical changes in the appearance of stimuli, other factors influence memory such as the encoding strategy used to study information. Over recent decades, bountiful evidence has shown that processing information in reference to the self has a strong influence on memory, a phenomenon known as the self-reference effect (Rogers et al., 1977; Symons and Johnson, 1997; Hartlep and Forsyth, 2000; Hamami et al., 2011; Leshikar and Duarte, 2012, 2014; Leshikar et al., 2015). Specifically, the self-reference effect is the improvement in memory for information (words, pictures) processed in reference to the self compared to control conditions. In typical self-reference experiments, participants are prompted to process information in relation to the self (“does this word describe me?”) or through other encoding tasks, such as semantic processing (“is this a common word in the English language?”) or processing features of words (“is this word written in upper case?”). Results show that processing words in reference to the self leads to superior memory compared to control conditions (Rogers et al., 1977; Symons and Johnson, 1997). Importantly, these self-reference investigations predominantly use explicit memory tests, which involve the conscious recollection of previously encountered information (such as recall/recognition tests). It is relatively unknown the extent that self-referencing might also influence memory as measured implicitly, even when participants cannot consciously remember having encountered materials before (Nelson et al., 1989; Roediger, 1990; Schacter et al., 1993). The few studies that have measured implicit self-reference effects, have done so in clinical populations (e.g., generalized anxiety, major depression; Bradley et al., 1995; Banos et al., 2001), and thus these past works are likely not generalizeable. When considering both explicit and implicit memory, there often exists a degree of dissociation (Nelson et al., 1989; Roediger, 1990; Roediger et al., 1992; Rajaram and Roediger, 1993; Schacter et al., 1993; Weldon, 1993), where variables that have an effect on explicit memory have limited or no observable effect on implicit memory (Jacoby and Dallas, 1981; Nelson et al., 1989; Rajaram and Roediger, 1993). For instance, Bassili et al. (1989) demonstrated that manipulations of the level of processing (deep vs. shallow processing) have no effect on implicit memory, but significant effect on explicit memory. Turning back to self-referential processing, since some work suggests that self-referencing is not simply a deeper level of processing but instead a “special” type of mnemonic processing (Kircher et al., 2000),^1^ it remains unclear whether processing information in relation to the self might lead to enhanced implicit memory (relative to control), in addition to the explicit memory enhancement frequently reported (Rogers et al., 1977; Symons and Johnson, 1997; Gutchess et al., 2007; Hamami et al., 2011; Leshikar et al., 2015). Thus, in this investigation we examined the extent self-referencing affects implicit memory. Finding an implicit self-reference effect would further showcase the powerful influence of self-referencing on memory.

Intriguingly, over the long history of the self-reference effect on memory, many investigations include a case condition where words are presented in upper or lower case (Rogers et al., 1977; Kendzierski, 1980; Derry and Kuiper, 1981; Klein and Kihlstrom, 1986; Gutchess et al., 2007). In the case condition of these experiments, participants are asked to judge whether words are presented in upper or lower case at study, which typically serves as a control condition. What is striking about this past work that uses such a case condition is that essentially no studies report memory as a function of case (upper, lower). Although these past studies have made important contributions to understanding self-referential processing, they also represent lost opportunity to gain insight into how physical changes to the appearance of words such as case (e.g., words written in upper case), in addition to self-referencing, might influence memory. In this study, we report memory as a function of case (upper, lower) to better understand how these characteristics influence subsequent memory, which may be useful knowledge in an era dominated by text communication.

Turning to how case might interact with self-referencing, a theoretical perspective suggests that processing self-relevant information has a strong influence not just on memory but also on other processes such as perception. Specifically, this work suggests that self-referencing enhances the integration of details of an episode which can include binding perceptual details together as well as binding information to their source (Sui and Humphreys, 2015; Humphreys and Sui, 2016). The idea that self-referencing enhances the binding of perceptual details is supported by numerous experimental works (Sui and Zhu, 2005; Keyes and Brady, 2010; Keyes, 2012; Cunningham et al., 2014; Leshikar et al., 2015). It may be then that perceptual details that attract attention, such as words written in upper case, may interact with self-referential processing leading to increased memory relative to stimuli that are less attractive to attention, such as words written in lower case (i.e., words written in upper case will show an even larger self-reference effect than words written in lower case). Alternatively, if self-referencing equally enhances binding of perceptual details, then it is also possible that there would be no difference in the effect of self-referencing on memory for upper and lower case words (i.e., main effect of self-referencing and main effect of case, but no interaction). Given that past self-referencing work has not focused on how case might interact with self-referential processing, this is currently unknown.

In this experiment, participants were shown words written in upper and lower case while making one of three judgments for studied words: self-reference, commonness (semantic control), and case judgment. Participants then completed an implicit memory test (word stem completion task), where participants were presented with the first two letters of a word and asked to report the first word that comes to mind (Rajaram and Roediger, 1993; Roediger and McDermott, 1993; Schacter et al., 1993). Because of our interest in examining how self-referencing might improve integration of perceptual details (especially whether words are presented in upper or lower case), we chose to use an implicit memory measure known to be attuned to changes in perceptual details (word stem completion; Roediger and McDermott, 1993). Participants then took an explicit memory test that measured item memory as well as memory for two different contextual details: *source context*, where participants judge in which condition a word was studied, and *case context*, where participants report whether words were presented in upper or lower case at study. We make three predictions in this experiment: the first two about explicit memory, and the third about implicit memory. First, for our measures of explicit memory, we predict a *case enhancement effect* where memory for words written in upper case will be better than words written in lower case. Such a finding would be in line with past work showing case enhancement effects, but under conditions more carefully controlled (equal presentation of materials in upper and lower case) than past work (Schulman, 1974; Lorch et al., 1995; although see Craik and Tulving, 1975; Foster and Coles, 1977; Hunt and Elliot, 1980). Second, we predict the standard self-reference effect in explicit memory for both *item* and *context* memory, consistent with prior reports (Hamami et al., 2011; Serbun et al., 2011; Leshikar and Duarte, 2012, 2014; Leshikar et al., 2015; Zhang et al., 2019). Finding a memory benefit for contextual details would further show that self-referential processing is a powerful mnemonic that improves explicit memory for multiple details associated with an episode. As for the interaction of case and self-referencing, we see one of two possibilities. It may be that self-referencing might serve to enhance processing of perceptual details that capture attention such as words written in upper case; however, it is also possible that variation in perceptual details like case (whether upper or lower) might be equally supported under self-referential encoding compared to control conditions. Either outcome is consistent with work showing that self-referencing enhances binding of perceptual details (Sui and Humphreys, 2015). Third, for implicit memory, we expect one of two outcomes with respect to self-referencing. If self-referencing *only* reflects a deeper level of encoding as some argue (Gillihan and Farah, 2005), we predict that there will be no effect of self-referential processing on implicit memory relative to control. Such an outcome would be consistent with findings that implicit memory is not affected by differences in processing depth (Bassili et al., 1989). Alternatively, if processing information in reference to the self is not simply a deeper level of encoding, but instead reflects processing information through the lens of a superordinate schematic structure that significantly improves memory, as others argue (Rogers et al., 1977; Kircher et al., 2000), then it is possible that we might see an increase in implicit memory for items processed self-referentially relative to control. Such a result would suggest that self-referencing has such a strong effect on memory that it can be measured implicitly as well as explicitly, and is not simply a deep level of processing. As for changes in the physical appearance of words, we do not expect that words written in upper case will affect implicit memory performance, in line with evidence that implicit memory is not strongly affected by the case (upper, lower) in which words are presented (Rajaram and Roediger, 1993). Overall, this work will allow us to examine the extent physical changes in word appearance and engagement in self-referential processing may influence memory as measured explicitly and implicitly.

## Materials and Methods

### Participants

Twenty-seven adults (average age = 19.7, *SD* = 2.5, age range = 18–22, females = 11) were recruited from the subject pool at the University of Illinois at Chicago (UIC). Participants were over 18 and fluent in English (with no other inclusion criteria). One participant was excluded due to below chance performance on all explicit memory measures. An *a priori* power analysis performed in G*Power (Faul et al., 2007) based on effect sizes from our past work (Leshikar et al., 2015) suggested that 17 participants would be sufficient to examine the memory effects of interest with power of 0.8 and an alpha at 0.05. Participants provided informed consent in accordance with the University of Illinois at Chicago Institutional Review Board guidelines. Each participant received course credit for their involvement in the study.

### Stimuli

A total of 96 adjectives taken from Anderson (1968), were used in this study. Words contained between 4 and 8 characters. Half the words were positive and half negative, as defined by average likeability ratings from Anderson (1968; positive mean = 459.2; negative mean = 165.6).^2^ Because of our use of a word stem completion task where we presented the first two letters of a word for our implicit memory measure, we constructed the stimuli set so that the first two letters of each word were unique (e.g., the word “happy” was in our set of stimuli, thus no other word started with the letters “ha,” etc.). Across participants, words were counterbalanced to appear as a studied item in either upper or lower case in each of the three encoding conditions (self-reference, commonness, case) or as a novel word at retrieval.

### Procedure

This experiment was divided into three phases: encoding, implicit retrieval, and explicit retrieval. Material in all phases were presented on a monitor and the participants were tested one at a time in a quiet room. All words were presented in white 36-point Arial font on a black background. Participants first completed a practice session for the encoding and explicit retrieval phases of the experiment to ensure participants understood task instructions. All stimuli were presented in E-Prime.

**Figure 1:**
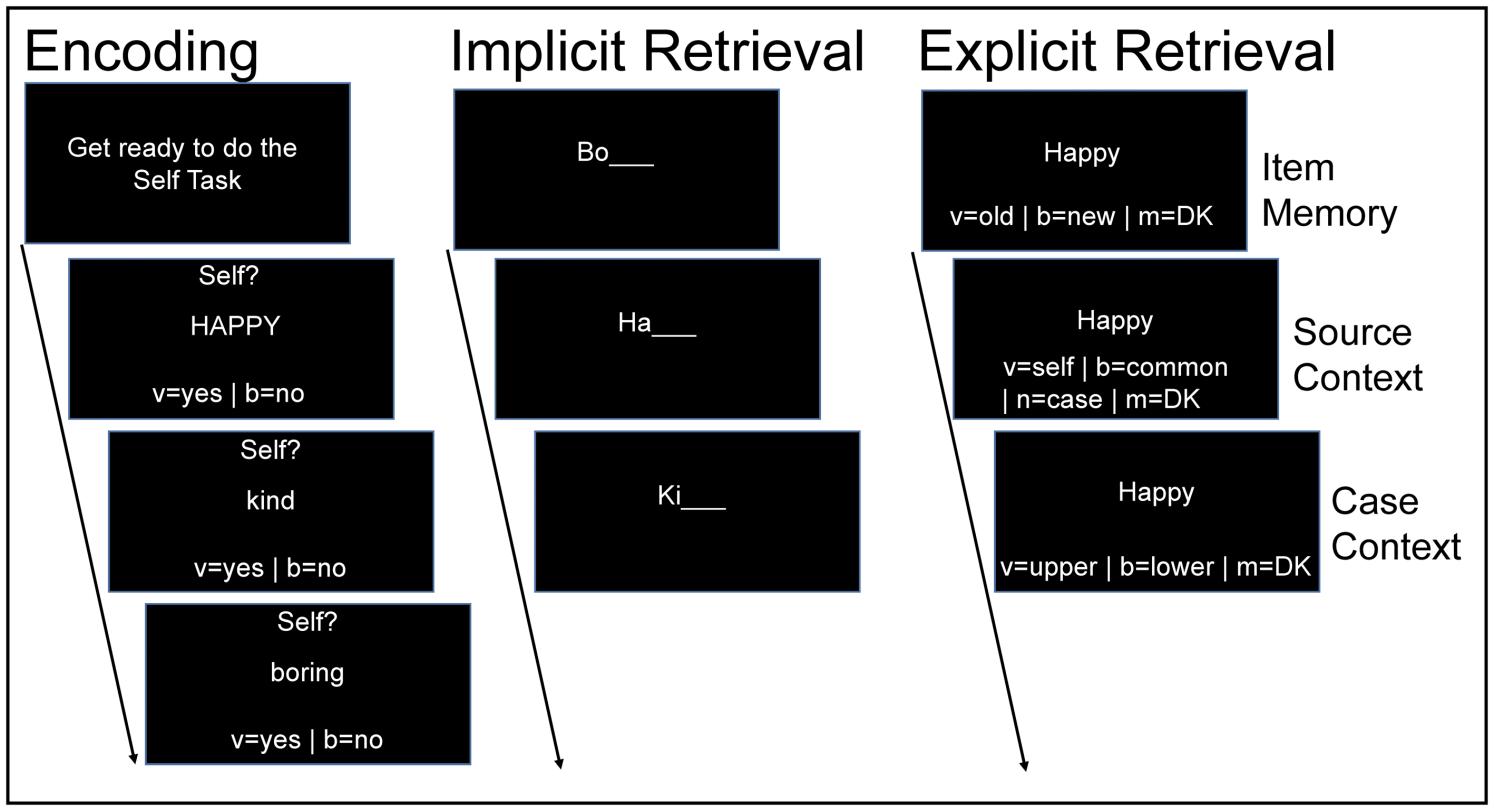
Trial depiction of the encoding, implicit retrieval, and explicit retrieval phases of the experiment. “DK” means “do not know.”

The encoding phase of the experiment began immediately after the practice session. Seventy-two self-paced trials were presented to the participants at encoding, with 24 words presented in each of the following conditions: self-reference, commonness (semantic control), and case. For each encoding trial, a word was displayed on the monitor in either upper or lower case letters and participants were prompted to make one of three judgments about that word (see Figure 1). In the self-reference condition, participant judged whether the word was self-descriptive (“does this word describe you?”), as we have done before (Leshikar et al., 2015). In the commonness condition, participants decided whether the word was commonly used in the English language (“is this a common word?”), and in the case condition, participants judged whether the word was presented in upper case (“Is this word written in upper case?”). Participants were instructed to press specific keys on the keyboard for each trial (v = yes | b = no), using the index and middle fingers of their right hand, respectively. Words were presented in mini-blocks of 12 consecutive trials of the same encoding task (e.g., self-reference, etc.). Before each mini-block, a notification was displayed for 6,000 ms (“Get ready for the Self/Commonness/Case Task”) alerting the participants of the upcoming task. Across participants, the order of mini-blocks was random with the constraint that two mini-blocks of the same condition (self-reference, commonness, case) were not presented consecutively.

After encoding, participants completed the implicit retrieval phase (word stem completion). Participants completed 96 self-paced trials in this phase. For each trial, a single word stem consisting of the first two letters of a word was presented (e.g., “Ha”). Seventy-two of the stems were from words studied at encoding, and 24 stems were new words. Participants were prompted to type the first word that came to mind by typing the missing letters to form a complete word. Word stems for studied words and new words (unseen at encoding) were presented in a random order.

After implicit retrieval, participants completed the explicit retrieval phase. Participants were shown 96 words (72 studied at encoding, 24 new), and were asked to make three self-paced recognition judgments for each word corresponding to item, source context, and case context memory, respectively. For each word, participants were first asked “Have you seen this word before?” to indicate whether they saw that word during the encoding phase of the experiment. Participants judged whether the word was old, new, or whether they did not know (v = old | b = new | m = DK) which served as our item memory judgment. Second, participants judged “In which condition did you see this word?” (v = self | b = common | n = case | m = DK) which was our source context memory judgment. Third, participants were asked “When you saw this word before, was it in upper case or lower case?” (v = upper case | b = lower case | m = DK) which served as our case context memory judgment. The “do not know” response option was included to minimize potential data contamination by guesses as done before (Duarte et al., 2008; Leshikar et al., 2015; McCurdy et al., 2017, 2019, 2020). All responses were made with the index, middle, ring, and pinky finger of the right hand. Because words at encoding were presented in either upper or lower case, we presented all words in title case where only the first letter of the word was capitalized. Studied and new words were presented in a randomized order.

## Results

We analyzed four measures of memory: item, source context, case context, and implicit. A 3 (Condition: self-reference, commonness, case) by 2 (Case: upper, lower) repeated-measures ANOVA with Condition and Case treated as within-subject variables was conducted for each of the explicit memory measures (item, source context, case context) as well as the implicit memory measure. All analyses were run in the JASP software package. Mean responses for the explicit and implicit memory measures are included in Tables 1–4.

**Table 1:** Item memory (explicit memory).

		Item memory response rates					
		Correct		Incorrect		Do not know	
Condition	Case	*M*	*SD*	*M*	*SD*	*M*	*SD*
Self-reference	Upper	0.83	(0.13)	0.14	(0.12)	0.03	(0.06)
	Lower	0.84	(0.13)	0.12	(0.10)	0.04	(0.07)
Commonness	Upper	0.70	(0.18)	0.25	(0.16)	0.05	(0.07)
	Lower	0.69	(0.17)	0.25	(0.16)	0.06	(0.09)
Case	Upper	0.36	(0.18)	0.56	(0.22)	0.08	(0.13)
	Lower	0.28	(0.13)	0.64	(0.15)	0.08	(0.12)
New words		Correct rejection		False alarm		Do not know	
		0.70	(0.17)	0.20	(0.14)	0.10	(0.11)

Proportion of correct, incorrect, and “do not know” responses as a function of condition and case for old words seen at encoding, as well as the proportion of correct rejections, false alarms, and “do not know” responses for new words. Our use of the term “Correct” to previously seen items is analogous to the term item memory hit. Similarly, our use of the term “Incorrect” for the previously seen items is analogous to the term item memory miss.

**Table 2:** Source context memory (explicit memory).

				Source context response rates					
				Correct		Incorrect		Do not know	
Condition	Case			*M*	*SD*	*M*	*SD*	*M*	*SD*
Self-reference	Upper			0.60	(0.26)	0.19	(0.16)	0.21	(0.21)
	Lower			0.67	(0.24)	0.15	(0.14)	0.18	(0.19)
Commonness	Upper			0.36	(0.18)	0.31	(0.18)	0.33	(0.21)
	Lower			0.42	(0.22)	0.28	(0.15)	0.30	(0.21)
Case	Upper			0.08	(0.10)	0.24	(0.15)	0.68	(0.19)
	Lower			0.07	(0.10)	0.19	(0.13)	0.74	(0.14)
New words		Self-Ref FA		Common FA		Case FA		Do not know	
		0.07	(0.07)	0.06	(0.06)	0.06	(0.07)	0.81	(0.15)

Proportion of correct, incorrect, and “do not know” responses as a function of condition and case for old words seen at encoding, as well as the proportion of false alarms (FA) and “do not know” responses for new words. “Self-Ref FA” is when a participant endorsed a new word as having occurred in the self-reference condition. “Common FA” is when a participant endorsed a new word as having occurred in the commonness (semantic control) condition. “Case FA” is when a participant endorsed a new word as having occurred in the case condition.

**Table 3:** Case context memory (explicit memory).

		Case memory response rates					
		Correct		Incorrect		Do not know	
Condition	Case	*M*	*SD*	*M*	*SD*	*M*	*SD*
Self-reference	Upper	0.32	(0.18)	0.37	(0.19)	0.31	(0.24)
	Lower	0.54	(0.25)	0.16	(0.17)	0.29	(0.26)
Commonness	Upper	0.26	(0.19)	0.30	(0.15)	0.44	(0.23)
	Lower	0.44	(0.23)	0.11	(0.13)	0.45	(0.27)
Case	Upper	0.08	(0.11)	0.16	(0.14)	0.76	(0.20)
	Lower	0.14	(0.10)	0.09	(0.11)	0.77	(0.15)
New words		Upper FA		Lower FA		Do not know	
		0.04	(0.07)	0.11	(0.11)	0.85	(0.16)

Proportion of correct, incorrect, and “do not know” responses as a function of condition and case for old words seen at encoding, as well as the proportion of false alarms, and “do not know” responses for new words. “Upper FA” is the proportion of new words falsely endorsed as having been presented in upper case. “Lower FA” is the proportion of new words falsely endorsed as having been presented in lower case.

**Table 4:** Implicit memory.

		Implicit memory response rates			
		Matched		Unmatched	
Condition	Case	*M*	*SD*	*M*	*SD*
Self-reference	Upper	0.08	(0.07)	0.92	(0.07)
	Lower	0.07	(0.06)	0.93	(0.06)
Commonness	Upper	0.09	(0.10)	0.91	(0.10)
	Lower	0.07	(0.07)	0.93	(0.07)
Case	Upper	0.05	(0.05)	0.95	(0.05)
	Lower	0.06	(0.08)	0.94	(0.08)
New words		*Matched*		*Unmatched*	
		0.03	(0.03)	0.97	(0.03)

Proportion of matched and unmatched responses as a function of condition and case for old words seen at encoding, as well as the proportion of matched and unmatched responses for new words. For words studied at encoding, “Matched” refers to instances where the word generated by the participant was the same as the word presented at encoding. “Unmatched” refers to instances where the generated word and the presented word at encoding differed. For new words, “Matched” refers to instances where participants produced the word in our stimulus set (despite not seeing that word at encoding), and “Unmatched” refers to when participants produced a word that differed from our stimulus set.

**Figure 2:**
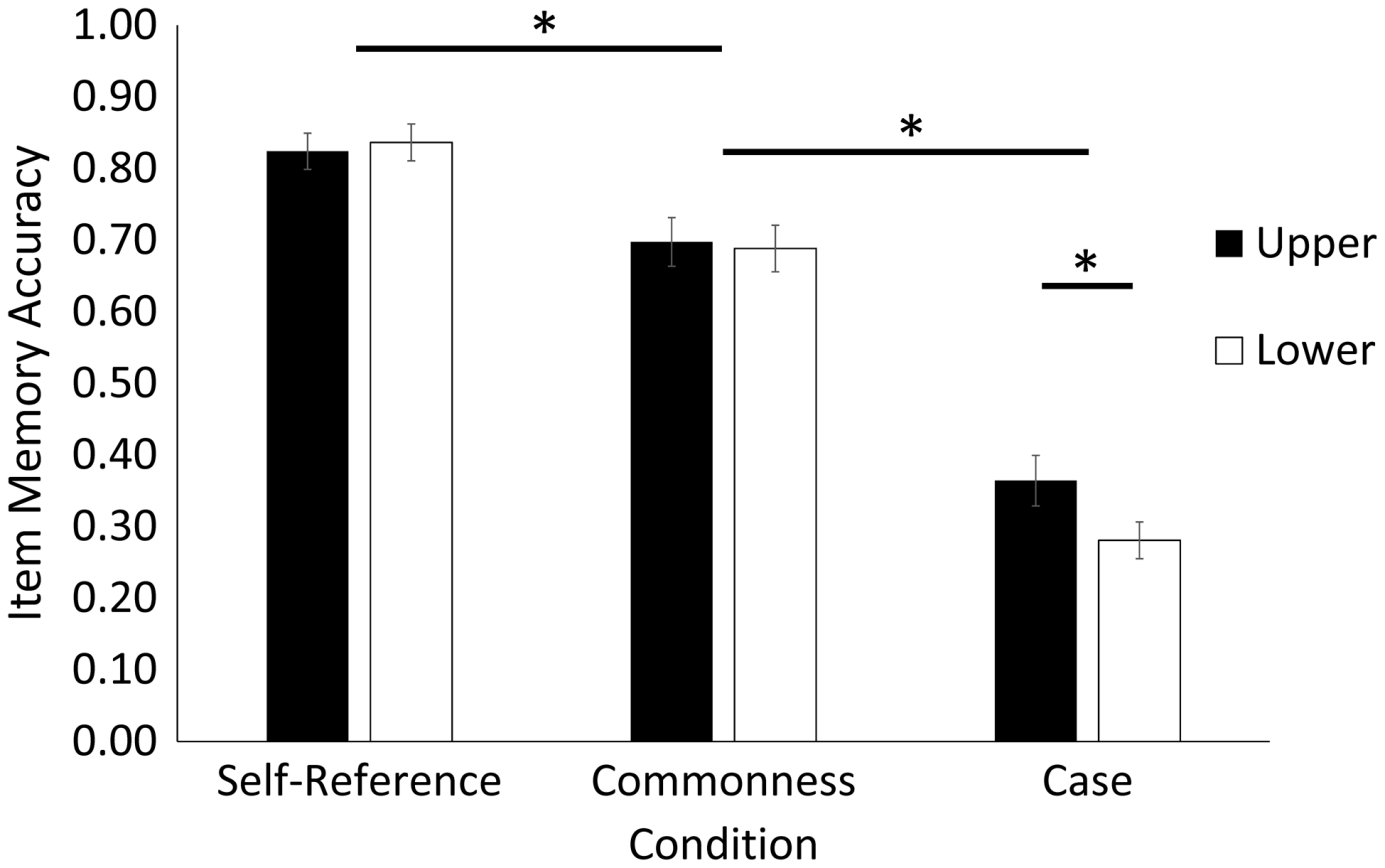
Item memory (explicit memory) as a function of encoding condition and case. Item memory results showed a self-reference effect, and further indicated a case enhancement effect for words presented in upper compared to lower case, but only in the case condition. Error bars represent standard error. Asterisks denote statistical difference at *p* < 0.05.

Item memory (explicit memory) was calculated as the proportion of old words correctly recognized out of the total number of old words presented to the participants. Item memory results are depicted in Figure 2. There was a main effect of condition, *F*(2, 52) = 186.67, *p* < 0.001, $\eta_{p}^{2}$ = 0.88. Paired sample *t*-tests indicated that item memory was better in the self-reference condition (*M* = 0.83, *SE* = 0.02) than in commonness (*M =* 0.69, *SE* = 0.03), *t*(26) = 5.05, *p* < 0.001, *d* = 0.97, and case conditions (*M* = 0.32, *SE* = 0.03), *t*(26) = 18.68, *p* < 0.001, *d* = 3.59. Additionally, item memory was better in the commonness condition than in the case condition, *t*(26) = 13.63, *p* < 0.001, *d* = 2.62. There was no main effect of case, *F*(1, 26) = 1.91, *p* = 0.179, $\eta_{p}^{2}$ *=* 0.07. There was, however, a condition by case interaction, *F*(2, 52) = 3.34, *p* = 0.043, $\eta_{p}^{2}$ = 0.11, which was driven by better memory for words presented in upper (*M* = 0.36, *SE* = 0.04) compared to lower case (*M =* 0.28, *SE* = 0.03), but only for words presented in the case condition, *t*(26) = 2.65, *p* = 0.014, *d* = 0.51. There was no difference in item memory between upper and lower case words in the self-reference *t*(26) = 0.40, *p* = 0.691, *d* = 0.08, and commonness, *t*(26) = 0.35, *p* = 0.729, *d* = 0.07, conditions.

**Figure 3:**
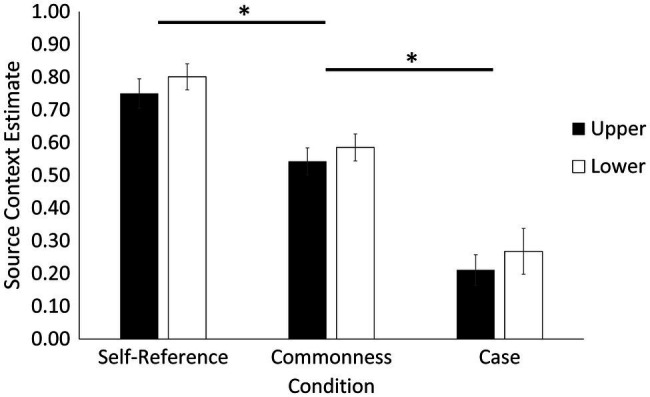
Source context memory estimates (explicit memory) as a function of encoding condition and case. Results indicated a self-reference effect, and a case effect where memory was better for words presented in lower compared to upper case across all conditions. Error bars represent standard error. Asterisks denote statistical difference at *p* < 0.05.

Second, we calculated a memory estimate for source context (explicit memory). This estimate was calculated as the number of times participants correctly remembered the condition in which the word was presented at encoding out of the number of times they made a source attribution [source correct/(source correct + source incorrect)], which is a conditionalized measure that removes the influence of item memory on context memory (Bayen et al., 1996) and is a measure used in related work (Leshikar et al., 2014; Leshikar and Gutchess, 2015; Leshikar et al., 2016). Source context results are depicted in Figure 3.^3^ Source context data showed that the sphericity assumption of repeated measures ANOVAs was violated, so we report sphericity corrected Greenhouse–Geisser statistics. The analysis revealed a main effect of condition, *F*(1.88, 45.02) = 52.76, *p* < 0.001, $\eta_{p}^{2}$ *=* 0.69. Follow-up *t*-tests indicated that source context memory was better in the self-reference (*M* = 0.78, *SE* = 0.04) compared to both the commonness (*M* = 0.57, *SE* = 0.03), *t*(25) = 4.02 *p* < 0.001, *d* = 0.81, and case conditions (*M* = 0.24, *SE* = 0.05), *t*(25) = 10.20, *p* < 0.001, *d* = 2.04. Source context memory was also better in the commonness than case condition, *t*(25) = 6.17, *p* < 0.001, *d* = 1.24. There was also a main effect of case, *F*(1, 24) = 5.38, *p =* 0.029, $\eta_{p}^{2}$ = 0.18, indicating that source context memory was better for words presented in lower (*M* = 0.55, *SE* = 0.03) compared to upper case (*M* = 0.50, *SE* = 0.03). There was no condition by case interaction, *F*(1.62, 38.87) = 0.02, *p* = 0.964, $\eta_{p}^{2}$ = 0.001.

**Figure 4:**
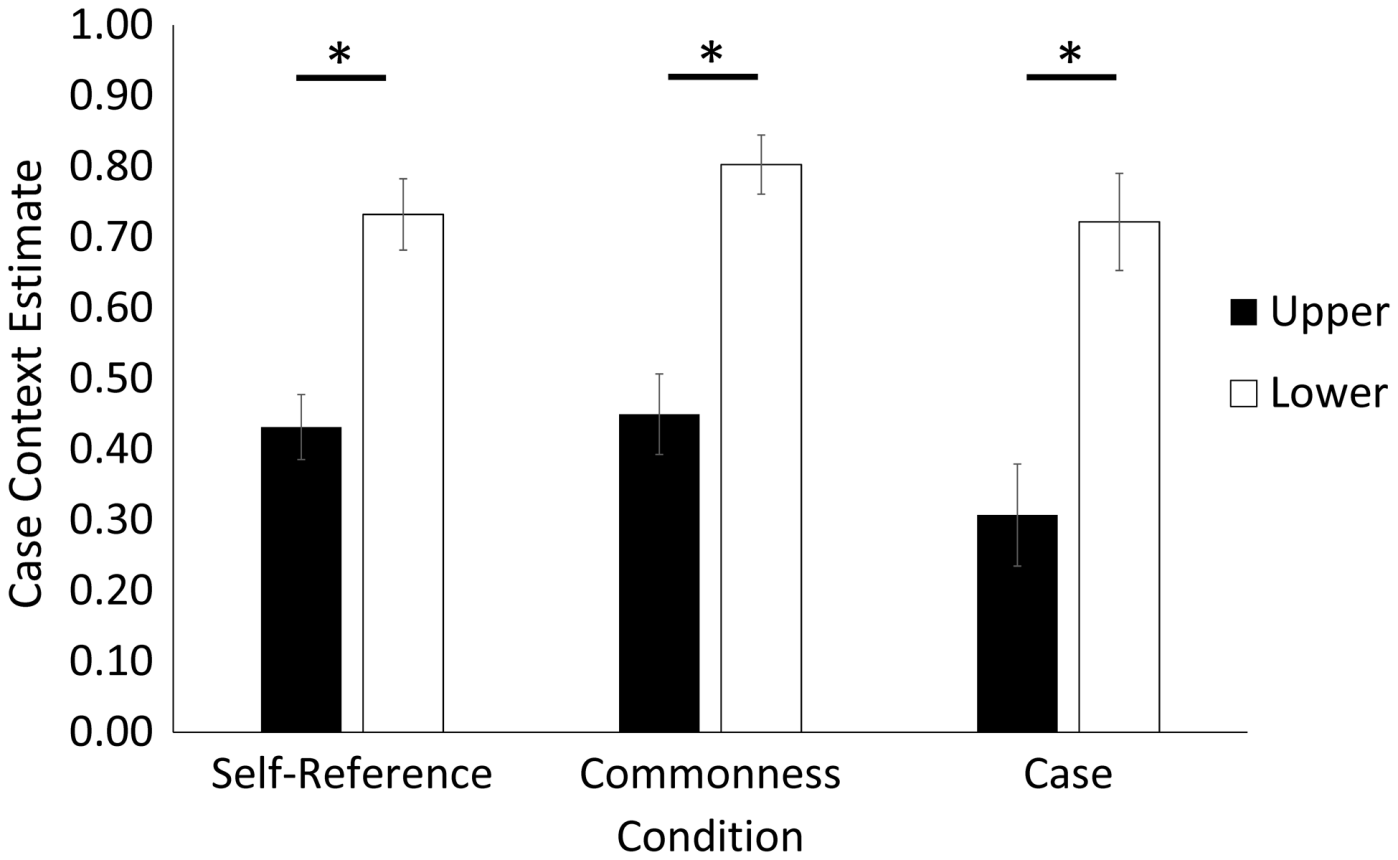
Case context memory estimates (explicit memory) as a function of encoding condition and case. Results showed a case effect where memory was better for words presented in lower compared to upper case across all encoding conditions. Error bars represent standard error. Asterisks denote statistical difference at *p* < 0.05.

For case context memory (explicit memory), we calculated an estimate analogous to that of source context. This estimate was calculated as the number of times participants correctly remembered the case in which a word appeared at encoding out of the number of times they made a case attribution [case correct/(case correct + case incorrect)]. Case context results are depicted in Figure 4.^4^ There was no main effect of condition, *F*(2, 42) = 2.74, *p =* 0.076, $\eta_{p}^{2}$ = 0.12; however, there was a main effect of case, *F*(1, 21) = 49.11, *p* < 0.001, $\eta_{p}^{2}$ = 0.70, showing that case context memory was better for words presented in lower (*M* = 0.75, *SE* = 0.03) compared to upper case (*M* = 0.40, *SE* = 0.04), which is not aligned with our predictions, but could imply a possible trade-off in memory for materials presented in upper case. There was no condition by case interaction, *F*(2, 42) = 0.57, *p* = 0.572, $\eta_{p}^{2}$ = 0.03.

Implicit memory was calculated as the proportion of times the word used to complete the word stem matched the word presented at encoding. Results showed no main effect of condition, *F*(2, 52) = 1.76, *p* = 0.181, $\eta_{p}^{2}$ = 0.06, case, *F*(1, 26) = 0.03, *p* = 0.873, $\eta_{p}^{2}$ = 0.001, or interaction, *F*(2, 52) = 1.18, *p* = 0.316, $\eta_{p}^{2}$ = 0.04. Because it is not possible to draw conclusions about null results from frequentist analysis approaches (e.g., traditional ANOVAs), we further examined these data using a Bayesian ANOVA to examine whether there was significant support for the null hypothesis relative to the alternative hypothesis (i.e., that self-referential processing would lead to improved implicit memory compared to control conditions). Results showed a Bayes Factor (BF01) of 6.50 for the comparison between the self-reference and commonness condition, suggesting that the null hypothesis was more than 6 times likelier than the alternative hypothesis. Past work suggests a Bayes Factor of between 3 and 9 suggest significant evidence in favor of the null (Jeffreys, 1998). Alternatively, results showed a Bayes Factor (BF01) of 1.61 for the comparison between the self-reference and case condition. Although we found no evidence of implicit memory effects across the studied items, past work suggests that there should be a measurable difference in implicit memory between old vs. new words. To check whether we observed this phenomenon in our data, we further conducted pairwise comparison between old items seen in the self-reference, commonness, and case condition against new words. Consistent with past work, participants completed word stems using stimuli presented in the self-reference condition (*M* = 0.08, *SE* = 0.01), commonness (*M* = 0.08, *SE* = 0.01), and case conditions (*M* = 0.06, *SE* = 0.01) at higher rates than new words (*M* = 0.03, *SE* = 0.01), *ts >* 2.11, *ps* < 0.044, *ds* > 0.41.

## Discussion

In this experiment, we evaluated the extent physical changes to stimuli (case) and self-referential encoding influenced both explicit and implicit measures of memory. There are three main findings in this experiment. First, we found a modest case enhancement effect where words written in upper case were better remembered than those written in lower case as measured by item memory, but only in the case condition when words were processed for case. This result only partially supports previous findings of a case enhancement effect (Foster and Coles, 1977; Lorch et al., 1995). Critically, this item memory case enhancement effect was accompanied by steep decreases to context memory, suggesting an item-context memory trade-off, where a slight increase in item memory was associated with context memory decreases. Second, we found a self-reference effect in explicit memory as measured by both item and source context memory, which is consistent with past findings (Hamami et al., 2011; Serbun et al., 2011; Leshikar and Duarte, 2012, 2014; Leshikar et al., 2015; Zhang et al., 2019), and further suggests that self-referential processing has a strong influence on episodic memory. Third, we saw no evidence that physical changes to words (case) or processing materials self-referentially had an effect on implicit memory as measured by word stem completion.

Previous research has demonstrated that presenting information in upper case improves explicit memory (Foster and Coles, 1977; Golding and Fowler, 1992; Lorch et al., 1995; Dyson and Gregory, 2002). Partially consistent with these findings, we found an item memory case enhancement effect for words presented in upper case, but only when participants were processing words in the case condition. Because past work on the effects of case on word perception suggests that words written in upper case may be more attention capturing (Berlyne, 1974), it is possible that the item memory case enhancement effect reflects added attention to words written in uppercase, but only when participants were oriented to the case of the word. We found no evidence, however, of a case effect in the self-reference or semantic control condition (commonness), which suggests that the influence of capitalization on memory is sensitive to encoding processes deployed by participants. Although speculative, it may be that when participants are engaged in encoding tasks that do not emphasize processing the case of the word (such as the self-reference or commonness condition) the effects of case on memory are negligible, if they exist at all, but when participants are oriented to the case, words written in upper case may be more attention capturing and thus remembered better compared to lower case words. Interestingly, much of the prior work on the beneficial effects of upper case formatting on memory have engaged participants in semantic processing tasks, where they are reading passages for meaning (Foster and Coles, 1977; Golding and Fowler, 1992; Lorch et al., 1995; Dyson and Gregory, 2002). In our study, we also induced semantic processing in the commonness condition. However, unlike the past typographical cueing work, we found no evidence of a case enhancement effect in the semantic condition. The fact that we did not see a case enhancement effect in our semantic condition suggests that there may be alternative explanations for these past reports of case enhancement effects. Indeed, much of this past work used experimental designs that involved embedding shorter capitalized texts in longer passages, which may have led the cued (upper case) materials to be more memorable simply because they were relatively distinctive (Hunt and Elliot, 1980), and not because of the capitalization itself. Thus, the work in this investigation brings into question the robustness and generalizability of the case enhancement effect on item memory seen before (Foster and Coles, 1977; Golding and Fowler, 1992; Lorch et al., 1995; Dyson and Gregory, 2002). Given that item memory in the case encoding condition was reduced relative to both the self-reference and semantic control condition, this further suggests that case enhancement effects, when they occur, are modest. In terms of implicit memory, our results showed that capitalization had no effect on implicit memory for the presented words. This finding is in line with past work demonstrating that changes to the case in which words are presented does not strongly influence implicit memory (Rajaram and Roediger, 1993).

Although we found some support for a modest case enhancement effect on item memory, we found a much stronger and more consistent effect of word case on context memory. Specifically, we found striking deficits in context memory as measured by both source and case context. The context memory effects were particularly evident for the case context measure where memory was substantially reduced for items presented in upper relative to lower case. It may be that items presented in upper case were effective in drawing attention to the item itself, but without leading to improved memory for case in which an item was presented. Finding evidence for modest item memory improvements with concomitant declines in context memory suggests an item-context memory tradeoff for materials written in upper case. Intriguingly, we found evidence of this context memory decline for all words written in upper case regardless of the condition in which the word appeared (self-reference, semantic control, case condition). This may suggest that seeing words written in all caps might sometimes make that content more memorable, but that it comes at a significant reduction to other contextual details that accompany the episode. Thus, the data in this experiment suggest that writing materials in upper case may not be an altogether effective strategy and may actually produce poorer memory for the cued text when considering both item and context memory together. One potential explanation for this item-context tradeoff finding could be that item and context memory require different processing mechanisms (Hirst, 1982), and thus, may compete for cognitive resources during encoding (Jurica and Shimamura, 1999). Consequently, an increase in the allocation of encoding resources to item memory may decrease the resources available for context memory (Zaragoza and Lane, 1994; Jurica and Shimamura, 1999), resulting in the observed enhancement in item memory and decline in context memory. Given that past work suggests that retrieving contextual details (as measured by context memory tests) is thought to be a more challenging retrieval task than making item memory recognition judgments (Spencer and Raz, 1995), this may have also contributed to the item-context trade-off we observed. Alternatively, another possible explanation for our context memory findings may be related to the nature of the context memory test we used. At explicit retrieval, we presented all words in title case where the first letter was capitalized, but the rest of the letters were in lower case. Because most of the letters in each word were presented in lower case at retrieval, this may have allowed participants to reinstate the encoding context for the words presented in lower case, which in turn may have allowed them to perform better on the context memory measures (for the words presented in lower case at encoding). We see this alternative explanation as less likely however, because if participants were successfully reinstating the encoding context more often for the word presented in lower than upper case, then this would likely have yielded substantially reduced “do not know” responses for the context memory judgments for the lower (compared to upper) case words (consistent with past work examining context reinstatement effects; Hanczakowski et al., 2014, 2015), which is not what we observed. Given the ubiquity of communication *via* text, our data suggest a significant downside to writing messages in upper case if the intent behind those messages is to convey memorable information. Stated differently, writing in upper case may not be an effective communication strategy if one intends to convey memorable information, because the modest increases in memory for the content of the cued text (i.e., item memory) may come at a cost to other episodic details (e.g., context memory).

Turning to the self-reference findings, we found a strong effect of self-referencing on explicit memory as measured by both the item and source context measures. There is abundant evidence that self-referential processing improves explicit memory relative to control conditions (Markus, 1977; Rogers et al., 1977; Kuiper and Rogers, 1979; Symons and Johnson, 1997; Sui and Humphreys, 2015). The fact that we found a self-reference effect for source context memory suggests that self-referencing is a powerful mnemonic which supports the encoding of multiple details (item and source) into a retrievable memory representation. This is congruent with previous work showing that self-referencing not only improves memory for the items themselves, but also for other episodic contextual details (Dulas et al., 2011; Hamami et al., 2011; Rosa and Gutchess, 2011; Serbun et al., 2011; Leshikar and Duarte, 2012, 2014; Leshikar et al., 2015; Hou et al., 2019; Zhang et al., 2019). Unlike the source context measure, however, we found no improvement in memory for case context, suggesting some limits to explicit memory improvements that result from self-referencing. When considering all three memory measures together (item, source context, case context), we found no evidence that case interacted with self-referential processing. Specifically, we found no evidence for improved memory for words written in upper case (compared to lower) when processed self-referentially in any memory measures. Given that a theoretical account suggests that self-referential processing induces heightened processing of perceptual details (Sui and Humphreys, 2015; Humphreys and Sui, 2016; Sui and Rotshtein, 2019), it may be self-referencing enhanced memory for case for words presented in both upper and lower case. Such a possibility is consistent with so-called “self-prioritization” effects that show that even lower-level perceptual details (e.g., Gabor patches) that are associated with the self are associated with enhanced visual awareness (Stein et al., 2016; Macrae et al., 2017, 2018).

Although there is extensive literature highlighting the influence of self-referencing on explicit memory, there is a dearth of work investigating this phenomenon on implicit memory. In this study, we investigated the extent self-referencing might influence a measure of implicit memory but found no evidence of such an effect. Indeed, none of the encoding tasks (self-reference, semantic, case) had a measurable effect on implicit memory, which is consistent with past work showing that differences in depth of processing do not strongly influence implicit memory (Graf et al., 1985; Bassili et al., 1989; Nelson et al., 1989; Rajaram and Roediger, 1993). The fact that we found no hint of a self-reference effect on implicit memory is consistent with the idea that self-referencing is simply a deeper level of processing as some researchers suspect (Gillihan and Farah, 2005) and not a “special” mnemonic as others have argued (Kircher et al., 2000). Interestingly, our finding that self-referential processing had no observable effect on implicit memory is further consistent with another line of work investigating self-referencing effects on recollection and familiarity. This work has shown that self-referential processing enhances recollection, but not familiarity (Conway and Dewhurst, 1995). Because familiarity is thought to be sensitive to implicit memory (Yonelinas, 2002), the work of Conway and Dewhurst (1995) and others (Leshikar et al., 2015) is further consistent with the notion that self-referential processing has no strong influence on implicit memory. It is worth noting that the implicit memory measure we used (word stem completion) is generally considered a “perceptual” implicit memory assessment, which is especially sensitive to changes to perceptual features of stimuli (Roediger and McDermott, 1993). Given our interest in memory effects related to physical changes to stimuli (case) in this investigation, our use of a perceptual implicit memory assessment was apt. It is possible, however, that if we used a more conceptual measure of implicit memory (such as a word association task, “what word is associated with ____”; Shimamura and Squire, 1984), our patterns of results may have differed. Investigating ways to promote memory is an important scientific effort (Giannakopoulos et al., in press; Sklenar et al., in press; Yonelinas, 2002; Jennings et al., 2005; Matzen et al., 2015; Leshikar et al., 2017; Leach et al., 2019; Frankenstein et al., 2020; McCurdy et al., 2020, 2021; Villaseñor et al., 2021), and the results of this investigation contribute to that endeavor.

In this investigation, we found evidence for item-context trade-offs for items presented in upper vs. lower case, and that self-referential processing enhanced memory as measured explicitly (but not implicitly); however, there are three limitations of this investigation worth mentioning. First, participants knew that there would be an explicit memory test following encoding, which means that participants were studying items under intentional learning conditions. Such intentional learning conditions may have affected memory results for both explicit as well as implicit memory measure (Eagle and Leiter, 1964; Pressley et al., 1987; Naveh-Benjamin et al., 2009). Future work should extend these findings by engaging participants in incidental learning conditions, where they are unaware of the memory tests. Second, implicit retrieval always came before explicit retrieval in this experiment. Because of this experimental approach, it is possible that the implicit memory test may have influenced performance on the explicit memory test for items that were correctly produced in the implicit memory test. We argue, however, that even if this was the case, such effects would be small since relatively few items were correctly produced in the implicit retrieval test. Future work could counterbalance the presentation of the implicit and explicit retrieval to extend these findings. Third, in both our implicit and explicit memory tasks, participants were shown cues presented in title case where the first letter was capitalized, but the remaining letter/s were not (e.g., “Ha___” in the implicit test, and “Happy” in the explicit test) which could have affected our results. Specifically, the principles of transfer-appropriate processing suggest that memory is enhanced to the extent that conditions at encoding overlap with conditions at retrieval (Morris et al., 1977; Blaxton, 1989; Graf and Ryan, 1990; Franks et al., 2000), and thus presenting retrieval cues more closely aligned with how materials were shown at encoding (such as exactly matching the case of words), may boost memory performance. Future work should replicate and extend the present study by using retrieval cues that more closely match how words are presented at encoding.

## Conclusion

In conclusion, the findings of this study demonstrated that presenting words in upper case increase explicit memory performance under certain circumstances, but comes at a substantial cost to context memory, suggesting a tradeoff in explicit memory between item and context. We further demonstrated that self-referencing has a strong effect on memory when measured explicitly, but not implicitly, offering evidence that the beneficial effects of self-referential encoding may be attributable to a deeper level of processing. Overall, this work suggests that physical changes to the appearance of presented material as well as differences in the cognitive processes engaged to study those materials has a strong influence on memory as measured explicitly, but not implicitly.

## Data Availability Statement

The raw data supporting the conclusions of this article will be made available by the authors, without undue reservation.

## Ethics Statement

This study involving human participants was reviewed and approved by University of Illinois at Chicago. The participants provided their written informed consent prior to completing this study.

## Author Contributions

EL, AG, MM, and HN designed the experiment. HN, GI, and AS collected and analyzed the data. All authors contributed to the article and approved the submitted version.

### Conflict of Interest

The authors declare that the research was conducted in the absence of any commercial or financial relationships that could be construed as a potential conflict of interest.

## Footnotes

^1^Evidence that self-referential processing is not “just” a deeper level of processing has largely come from neuroimaging evidence showing that a different network of brain regions is active for self-focused compared to other types of deeper semantic processing.

^2^In Anderson (1968) the full range of possible scores for the likeability of words was from 0 (least likeable) to 600 (most likeable).

^3^For two participants, we were unable to estimate source context memory accuracy for the case condition which means these two participants had missing cells to run in our ANOVA. Because of this, data from these participants was excluded from analyses.

^4^For five participants, we were unable to estimate case context memory accuracy for the case condition which means these participants had missing cells and thus were excluded from analyses.
